# What Are Healthy Societies? A Thematic Analysis of Relevant Conceptual Frameworks

**DOI:** 10.34172/ijhpm.2023.7450

**Published:** 2023-11-07

**Authors:** Kent Buse, Amy Bestman, Siddharth Srivastava, Robert Marten, Sonam Yangchen, Devaki Nambiar

**Affiliations:** ^1^The George Institute for Global Health, Imperial College London, London, UK.; ^2^Faculty of Medicine, University of New South Wales, Sydney, NSW, Australia.; ^3^The George Institute for Global Health, New Delhi, India.; ^4^The Alliance for Health Policy and Systems Research, World Health Organization (WHO), Geneva, Switzerland.; ^5^Prasanna School of Public Health, Manipal Academy of Higher Education, Manipal, India.

**Keywords:** Healthy Societies, Social Determinants of Health, Structural Determinants of Health, Commercial Determinants of Health, Health Paradigms, Health Policy

## Abstract

**Background:** While support for the idea of fostering healthy societies is longstanding, there is a gap in the literature on what they are, how to beget them, and how experience might inform future efforts. This paper explores developments since Alma Ata (1978) to understand how a range of related concepts and fields inform approaches to healthy societies and to develop a model to help conceptualize future research and policy initiatives.

**Methods:** Drawing on 68 purposively selected documents, including political declarations, commission and agency reports, peer-reviewed papers and guidance notes, we undertook qualitative thematic analysis. Three independent researchers compiled and categorised themes describing the domains of a potential healthy societies approach.

**Results:** The literature provides numerous frameworks. Some of these frameworks promote alternative endpoints to development, eschewing short-term economic growth in favour of health, equity, well-being and sustainability. They also identify values, such as gender equality, collaboration, human rights and empowerment that provide the pathways to, or underpin, such endpoints. We categorize the literature into four "components": people; places; products; and planet. People refers to social positions, interactions and networks creating well-being. Places are physical environments—built and natural—and the interests and policies shaping them. Products are commodities and commercial practices impacting population health. Planet places human health in the context of the ‘Anthropocene.’ These components interact in complex ways across global, regional, country and community levels as outlined in our heuristic.

**Conclusion:** The literature offers little critical reflection on why greater progress has not been made, or on the need to organise and resist the prevailing systems which perpetuate ill-health.

## Background

 Improvements in social and economic conditions were responsible for significant population-level mortality declines in the 20th century.^1^ This led to an understanding that good health can be supported or inhibited by broader social circumstances. This resulted in social organising, policy efforts and research to prevent ill-health and promote health equity through collective action on structural determinants.

 Over the past 45 years, initiatives such as the Declaration of Alma Ata,^2^ the Ottawa Charter on Health Promotion^3^ and the Commission on Social Determinants of Health (SDoH)^4^ served as seminal moments to address the underlying conditions that lead to healthy societies. Yet, the predominant approach to creating health remains biomedical; focused on healthcare and treatment. Various analysts provide evidence, particularly in high-income settings, that the impact on health from medical care is limited^5^; there are estimates that at least 80% of health is related to socio-economic status, the physical environment and health behaviours.^6,7^ Within the health sector there is an underinvestment in preventing ill-health. Estimates suggest less than 10% of health spending is allocated to prevention.^8,9^ Societal efforts to prevent ill-health, enhance well-being and achieve social justice are neglected; consequently, health inequities continue to grow.

 The World Health Organization’s (WHO’s) 13th Global Programme of Work set three “one billion people targets,” one of which is to ensure one billion more people can enjoy better health.^10^ Under WHO’s present leadership, a new division has been created focusing on Healthier Populations, including departments dedicated to environment and climate change, SDoH, food safety and nutrition as well as health promotion. Yet compared to the two other one billion targets (on universal health coverage and health emergencies), this third billion is under-invested and under-studied. There is little clarity or agreement on the meaning of the terms “Healthier Populations” or “healthy societies,” its conceptual terrain, or its action and research agendas, even as similar debates are underway across regions of the world.^11^ This limits efforts to develop and implement relevant policies.

## Aims

 This mapping and framework building exercise does not respond to a narrow research question but aims to broadly understand how several linked concepts inform a broad range of efforts widely considered or conceptualized as efforts to create healthy societies. It is motivated by the hypothesis that a mapping of relevant literature could improve future research, political engagement and eventual policy interventions. This research did not seek to develop a unifying theory, but a heuristic that enables a descriptive analysis of “what” constitutes healthy societies. We also sought to review critically what was and was not included in these conceptualisations and the implications of these formulations for progress on this agenda. In another linked analysis, we sought to describe “how” the same literature proposes that healthy societies may be achieved.^12^

## Methods

 This study was initiated following conversations that senior authors were involved with on defining the scope of healthy societies. These interactions included some aimed at articulating what the WHO’s third “Triple Billion” target^10^ would encompass. Alongside this, some co-authors were involved with an institutional strategy-building on societal determinants of health and on articulating a research vision for “healthier societies.” These discussions were the starting point of our document selection which itself emanated from two reviews — namely Maani et al^13^ (reviewing how the commercial determinants of health [CDoH] are represented in social determinants frameworks) and van Olmen et al^14^ (a review of health systems frameworks).

 Our initial sample included purposively selected English language political declarations, commission and United Nations (UN) reports, peer-reviewed papers, commissioned evidence reviews and non-governmental organisation guidance notes (Supplementary file 1). Using the two initial review papers^13,14^ as a base, we determined inclusion criteria starting from the 1974 Lalonde Commission Report,^15^ as this report reinvigorated discussion around McKeown’s hypothesis about the determinants of health and marked a turning point in the global discourse.^16^

 Additional documents were identified using Google Scholar searches (using terms “Healthier Societies + Framework,” “Health + Framework,” “Health + Societies,” “Healthier Societies + approach,” “Health Systems + Framework”) covering the period 1974 to 2022. Through snowball sampling an additional 45 documents were proposed by the senior authors based on discussions during the analysis which were thought to be relevant to understanding the concept of “healthy societies.” We initially screened 202 documents using title and abstract details, and extracted data from 97 documents. We chose to exclude documents focussed on healthcare systems that added little to the discussion on keeping people out of such systems.

 Data about each paper were extracted into a coding template (Supplementary file 2). After reviewing extracted data, following consensus among co-authors, documents focusing on healthcare systems alone or those that do not add to the discussion on keeping people healthy and out of the healthcare system were excluded. Ultimately, 68 documents were used as the database for extraction (12 documents from the original review papers,^13,14^ 31 documents from Google Scholar search and 25 documents from snowballing or author suggestions). A PRISMA-ScR (Preferred Reporting Items for Systematic reviews and Meta-Analyses extension for Scoping Reviews) flowchart detailing this process is presented in Supplementary file 3. Additionally, in our paper, we draw on related publications to inform the analysis.

 Three researchers extracted data from included documents, initially independently followed by code-checking and discussions with two senior authors. Initial analysis, of both peer-reviewed and grey literature together, focused on basic descriptive details (date published, authors, affiliations, type of document, funder) and analytical information (aim, broad topics, policy approaches, and action and research agendas). Following initial analysis, a thematic approach was used for analysis. The findings focus on the presence of key themes, rather than the number of documents that refer to specific themes. Given the variation in our sample and our aim to explore the “what” of healthy societies, rather than the strength of evidence, a quality assessment of included documents was not conducted.

 Through an iterative process that included reviewing the extracted text in relation to different codes, noting themes and tabulations and reflecting on the implications, a heuristic was inductively developed to structure the presentation of the material (Figure). As data analysis progressed, this heuristic evolved. This paper focuses on the findings of the two inner parts of the heuristic: values and components. Levers, defined as instruments used by governments to elicit system-wide and societal change to meet objectives and/or respond to key stakeholders,^17^ and “enablers” are discussed in a companion paper on how to create healthy societies.^12^

**Figure:**
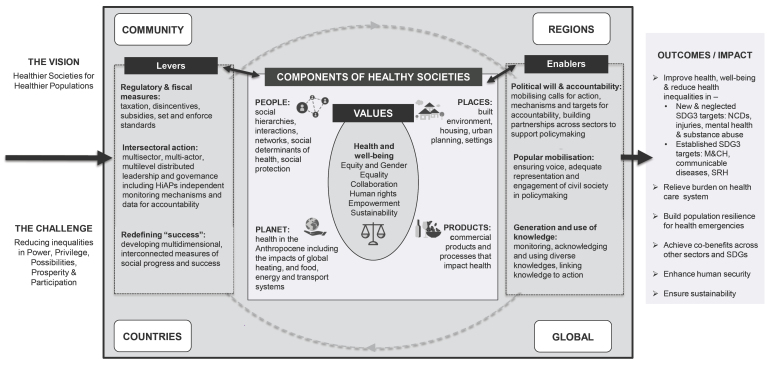
Heuristic of Healthy Societies. Abbreviations: HiAPs, Health in All Policies; M&CH, maternal and child health; NCDs, non-communicable diseases; SDGs, Sustainable Development Goals; SRH, sexual reproductive health.

 Guiding principles or frames through which frameworks/authors view the world and/or promote health were extracted. These are considered as “values.” Values were included where specifically identified in the document or if they constituted a fundamental guiding concept. Based on an initial inductive search of documents for values, a coding template of values was developed and applied to the sample. Values were often mentioned in a preambulatory fashion; however, we included them in the analysis only if they were elaborated in a substantive manner. We do not report on how authors defined these values.

 The “components” in the heuristic were identified, defined and elaborated through an iterative process. Documents were categorised inductively. From this broad list, topics were grouped, discussed and then recategorised. As higher-level themes were identified we drew links between key themes and ultimately identified four related components: people; places; products; and planet. For each component illustrative examples extracted from the papers are presented.

 The findings section describes the heuristic and presents the values and each of the components. This is followed by a discussion that reflects on the frameworks, the positionality of the literature and the way the selected literature engages with wider paradigms. After considering limitations, the paper concludes with reflections.

## Results

###  Elaborating a Healthy Societies Heuristic 

 Figure presents a heuristic derived inductively from the included literature. The literature, particularly related to the social determinants, describes a range of inequalities across societies — pertaining to the political, social and/or economic position of individuals, groups, institutions, countries — which drive health inequities (these challenges are listed on the left of Figure). Outcomes are diverse and include improvements across the health and well-being related Sustainable Development Goals (SDGs) targets as well as building population resilience for health emergencies, and fostering sustainability. Societal inequalities shape the afore-mentioned outcomes through many pathways — in what we term the “black box”^[1]^ of healthy societies.

 Inside the healthy societies’ black box, the relationships between the components and levers and enablers can transform (or not transform) inputs to outcomes. For instance, relationships among people relate to the concepts of social capital and social cohesion.^4^ Relationships between people and the planet reflect the intertwined nature of population health and environmental sustainability (which can disproportionately harm the health of already disadvantaged populations worsening inequity).^19^ Similarly, the extent to which society allows powerful groups to finance, produce, promote and lobby for the consumption of health-harming products (ie, CDoH) impacts people’s health and well-being.^20^ The final set of fields relate to the interplay between different geo-political levels: communities, countries, regions and global — reflecting that components and levers interact across levels. Some documents focus on one level,^21,22^ while others cover several^23^ (Table S1, Supplementary file 1).

 While the fields within the heuristic are presented as discrete, there are many overlaps and linkages. For example, some environmental frameworks view “the environment and its ecosystems as socially determined”^24^ (this reflects the linkage between people, places and planet in our heuristic). It is for these reasons we sought to elaborate a heuristic that integrates and elucidates these connections across often linked concepts.

###  Values

 We situate values at the centre of the black box. Values drive relationships between the “components” and influence which policy levers are pulled and how hard. Values both reflect and are integral in shaping social norms, and point to the needed cultural shifts towards healthy societies.^19,25-28^ For example, overcoming the “empathy failure” described in the Lancet Commission on Planetary Health.^29^ The analysis identifies 21 values in the sample (Supplementary file 4). We focus on the six that appear most frequently. These values contrast with those that give rise to growing health inequities — in part, presumably, due to inadequate emphasis (or value) that has historically been given to healthy societies’ values in health policy and practice.

 Many frameworks position health, well-being, and even happiness as among the highest of societal values. For example, New Zealand complements economic growth with the value of well-being in its 2019 budget^30^ and Bhutan position gross national happiness as the endpoint of development.^31^ However, critics have noted that the latter framework has overlooked human rights.^32^ In 2013, drawing on the “Stiglitz-Sen-Fitoussi” Commission on metrics for well-being and previous Human Development Reports,^33,34^ the WHO Europe Regional Office shifted from a focus on disease to one encompassing well-being, and identifies one of six 2020 targets as enhancing the well-being of Europeans.^20^ In short, several frameworks seek to elevate the societal value of well-being.

 The value identified most frequently is equity (in just over half of the documents). Throughout these texts, equity is closely related to the values of social justice and fairness.^3,4,35-39^ Tackling inequity features across the literature over the period under review.^3,4,40,41^ This includes guidance to incorporate equity-based principles in socio-economic policies that influence health^42^ (for example, on fair wages, taxation, poverty alleviation, or social insurance), calls for equitable distribution of health-creating resources and a general concern with health equity.^43^ Given that equity is a comparative principle, or judgment about how a person or group of people are situated relative to others, it is intriguing that few, if any, frameworks are explicit about underlying theories that provide for sense making of what is avoidable, unfair and unjust.The absence of clear guidance on determining how conditions are unjustly produced, raises questions about how this value may be translated into practice. Generally speaking, the frameworks would benefit from greater operational guidance to inform policy and practice on equity.

 Another prominent equity-oriented value is gender equality.^44-46^ Gender inequality interacts with a range of other social and economic determinants of health and is described as a driver and consequence of health inequities.^19,41,47^ For example, gender inequality experienced in early years can impact on agency and empowerment in adulthood.^45^ While documents refer to the need to reduce gender inequalities,^10,40,48^ only a small number considered specific actions such as addressing the gender pay gap, or equalizing parental leave to reduce gender inequality and the gendered determinants of health inequities.^22^ While older frameworks largely ignore gender considerations,^3,15,39^ newer ones recognise, for example, the differential impacts of climate change on different genders.^29^

 The value of collaboration, as well as the value of authentic partnership, is prominent across the sample.It is most evident in scholarship and commitments to all-of-government and all-of-society approaches.^49,50^ Collaborative mind-sets are crucial for people working across ministries, disciplines and the private sector as well as for effective community–researcher — policy-maker engagement, which is seen a driver of healthy societies.^50-53^ Despite this, in the sample, there was a distinct lack of research collaboration between the Global North and South. Of the 57 papers that listed author and affiliations in our sample only five documents included affiliations from authors from low- and middle-income countries (LMICs),^28,29,54-56^ comprising 23 (10%) of the 225 author affiliations. A quarter of documents were from international or regional groups that did not identify specific authors such as the People’s Health Movement or the Ottawa Charter.^3,46^ As with equity, collaboration features across the heuristic, for example, in the governance lever,^52,53^ and through calls for collective action related to political mobilisation.^3,23,29,57-59^ In contrast, to the value of collaboration, the literature offered less on the role of contestation, disruption and resistance in overcoming the status quo to foster healthy societies.

 While less prominent than other values, human rights and rights-based approaches are identified in many frameworks to ensure fairness, dignity and accountability^22,60^ (Supplementary file 4). The Alma Ata Declaration describes health as a fundamental human right.^2^ Several frameworks called for human rights to underpin actions for healthy societies and that they be protected through laws and fulfilled through a range of policy levers and enablers across multiple sectors.^3,28,61-63^ Several human rights are invoked including to development, participation, and respect for human dignity.^64^ Fox and Meier urge more attention to the collective right to development (rather than individual-oriented approaches), among other things“to bring about greater justice and fairness in economic relations between rich and poor countries.”^64^ Calls are made for the right to meaningful representation of a variety of civic groups in decision-making.^20^ More recent documents call for greater attention to rights to improve equity in relation to specific health outcomes,^64^ such as non-communicable diseases^23,28^ and COVID-19.^40^ Critics have called out the rights “blind spots” of some frameworks, arguing that the role of international conventions and rights-based responsibilities of nation-states are not sufficiently addressed by those working on the SDoH.^45^ Similarly, Givens et al review of 27 frameworks on health and equity, find only two explicitly mention the rights-related concerns of prejudice and stigma.^65^

 Empowerment refers to a person’s ability and power to act on behalf of themselves, and more broadly the opportunity to create “genuine possibilities” for such a situation.^60^ This value also encompasses collective empowerment for healthy societies.^20,23,59,60^ The structural capabilities that enable empowerment (or agency) are tied to policies and determinants (such as liberties, rights, income, wealth and resources) that enable “well-being” but also, the “freedom to pursue well-being.”^66^ While some authors note considerable ambiguity surrounding the concept,^67^ the suggestion is that more empowered individuals have control over the factors that influence their health and well-being.^59^ In the context of planetary health, the United Nations Development Programme describes the contingency of individual agency, stating “people can be agents of change if they have the power to act.”^19^ Sen’s work around capabilities provides additional context for arguments for empowerment as a key value for societal well-being.^68^ Rather than examining the “well-ness” of an individual, Sen considers an individual’s success in pursuit of their “actual freedom to live well and be well.”^68^ A small number of documents in our sample identify empowerment as critical to the realisation of other values, such as equity and human rights.^3,20,23,60^

 The value placed on sustainability increases over the period concerned with more recent documents emphasising the role of the planet as the life support system for the health and future generations’ survival — and is given further impetus with attention to planetary health.^28,29^ However, sustainability is not linked solely to the environment, some frameworks consider it in relation to the sustainability of government programmes. For example, Cerf calls for sustainable infrastructure, particularly in LMICs.^54^ Similarly in COVID-19 recovery efforts, the UN asked how research can be better designed to foster sustainability.^40^ Others advocate for investment in social capital alongside structural interventions to ensure sustainability.^69^

 Values such as equity, collaboration and empowerment have been championed as critical to healthy societies. These values, as well as those listed in Supplementary file 4, imbue healthy societies with a moral compass. Nonetheless, a critical reflection on the treatment of values in this literature reveals that it is silent or does not question the prevailing values promoted in mainstream society, namely of neo-liberalism, personal responsibility, rejection of the nanny state, the primacy of efficiency and markets, etc — in other words it implicitly legitimises the hegemony of prevailing grossly unfair distribution of power and protection of economic interests that undermine each component of healthy societies.^70^

###  Components of Healthy Societies

####  People

 Building upon the work of Wilkinson and Marmot,^71^ the Commission on the SDoH identifies the role of people’s social environment in creating “*flourishing societies.*”^4^ As per this literature, we use the term “people” to encompass people’s positions in social hierarchies, the interactions, interdependencies and social networks among them, and the social environments in which interactions occur. How societies treat people shapes whether and how societies flourish. Across the sample, a focus on how people are empowered to engage in policy making for healthy societies is apparent,^2,3,46,52,59^ yet the field has been largely apolitical and atheoretical.^72^

 Peoples’ social environments cover a range of conditions (such as living, networks, socio-economic and social protection) that affect health.^4,44^ In 1974, Lalonde argued that health is influenced by factors beyond an individual’s control.^15^ Subsequent frameworks emphasise improving health equity by focusing on upstream and distal determinants.^48,73,74^ These factors include the distribution of wealth and income, legal status (eg, migrant) and educational opportunities.^41,42^ Others draw attention to personal characteristics such as race, gender and forms of discrimination impacting on health equity.^42,44,47^

 A focus on people draws attention to impacts of societal inequalities on health outcomes over the life-course.^10^ This approach reveals opportunities to intervene in critical windows of vulnerability and transition such as early child development^42,45^ or during adolescence^75^ as well as the role of policies on future health outcomes, such as policies that impact on access to formal employment, income equality, parental leave and access to early childhood education.^42,45^ At the other end of the life course, a range of other social determinants, such as access to social care, living alone,^27^ impact on older people’s health.^77^ Actors outside the health sector are critical in addressing inequalities across the lifecourse that can improve health equity and enable healthy societies.^4,45,76^

 Some of the literature considers the intergenerational features of healthy societies,^29,30,40,42^ reflecting on how societal choices and policies adopted by one generation may impact the health of succeeding generations.^20^ For example, the Lancet Commission on Planetary Health is stark: “we have been mortgaging the health of future generations to realise economic and development gains in the present.”^29^ Dyck draws attention to the influence of culture and tradition, (and loss of knowledge due to colonization) on the health and well-being of current and future generations of Aboriginal people^78^ given the relationship between the environment, culture and health. More recent reports call for indigenous and traditional knowledges to inform action on healthy societies.^28,79^

 The literature argues that healthy societies cannot occur without “comprehensive” strategies to address people’s needs more holistically.^15,38,80^ Some authors focus on identifying ways to address social determinants of specific health issues. For example, Friel and colleagues^81^ review evidence on inequities in healthy eating. They argue that strategies must cover: (1) governance structures; (2) policies directly influencing the food environment; (3) macroeconomic and social policies; (4) cultural and societal norms and values; and (5) daily living conditions.^81^ In other words, a range of strategies are required to make healthy choices possible or easier for people. Other authors identify the need for comprehensive strategies to address the mental well-being of children and adolescents,^75^ physical activity,^82^ tobacco use,^26^ alcohol consumption,^25^ and other health issues.

 Several frameworks examine the impacts of social capital and social cohesion.^20,83-85^ Recognising that social capital does not occur in a “vacuum,” Kawachi et al call for investigation into power and the structural forces that shape social capital.^69^ While there is some treatment of collective action through framing of concepts like social capital,^35,69^ we found that the literature continues to focus on behaviour at the individual level and only recently have nudge or population level interventions been brought to the fore. Many frameworks call for greater civic engagement, for example in governance, policy making and research,^20,86^ but less attention is given to how to support them to do so or ensure accountability for systems to enable it.

####  Places

 The Shanghai Declaration on Health Promotion states that “health is created in the settings of everyday life.”^58^ “Places” include the physical environments where individuals live, work, commute and play. Much literature focuses on the health impacts of the built environment^49,53,84,87^ (including housing),^4^ urban planning (including active transport^49,53,84^ and access to green spaces^88^) and environmental exposures.^49^ Some explore how institutionalised racism, sexism and social class impact on the nature of built environments, who inhabits them, and how this affects health.^49^

 The reviewed literature also covers settings-based interventions.^24,54,73,89^ Housing and workplaces show the most promise to reduce health inequalities, however the literature notes a gap on the impacts of transport, education and workplace policies on inequality.^89^ Sampled papers examine the impact of housing on health-related outcomes such as early childhood development,^85^ general well-being^22^ and access to nutritious food, education, and healthcare.^42^

 The Commission on SDoH^4^ asserts that “safe, secure, and fairly paid work, year-round work opportunities, and healthy work–life balance” are required. A Knowledge Network supported the Commission and its report drew attention to power relations across government, markets and labour as it affects health inequities and recommended taking a rights-based approach to decent work.^90^ Other workplace factors identified included exposure to material hazards, work-related stress and health damaging behaviours (such as smoking) within workplaces,^4^ as well as the role of work insecurity, such as temporary contracts or part-time work.^4,40,61^

 A key concept related to the places component of healthy societies is attention to inclusive “systems approaches” to address environment-related burdens of disease by hardwiring health impacts into the planning departments of housing, transport and energy^53^ and collaborative decision-making by planning and health sectors.^50^ Better identification and measurement of environmental inequalities could promote access to green space, opportunities for active transport,^49,77^ zoning laws that address social inequalities^49^ and reducing environmental hazards such as air pollution.^15,85^ This literature stresses equity considerations as critical in guiding urban planning processes, including equity between rural and urban and within urban settings,^4^ and argues for incentives for developers to better meet the needs of low-income communities.^49^ Approaches focusing on specific health outcomes (eg, childhood development^45^ and childhood cancer^43^) similarly call for more coordinated approaches to address intersecting built, social and economic environments.

 The literature highlights the interdependencies between interventions focusing on people and places.^53^ Frameworks dealing with places tend to address urban settings in high-income income countries.^45,52,60^ It has also tended to focus on physical spaces, yet important emerging places for the creation of health and illness are now also found in virtual settings.^40^ The role of ownership of spaces, related to privatization of the commons and of resources that affect well-being is relatively underdeveloped. Tenure affects access to several social resources, be it in the rural context, or in the context of the urban poor. There was relatively limited consideration of this in the literature.

####  Products

 The term “products” is used as shorthand for commodities that improve health and those that create ill-health. These CDoH also include the practices and attributes of commercial entities (including financing, extracting, producing, marketing, and distributing these products). As early as 1986, the Ottawa Charter recognised the need to “counteract the pressures towards harmful products,”^3^ presenting the first reference to these determinants in our sample.

 Most documents in our sample focus on products (or industries) responsible for ill-health and the required but often inadequate regulatory environments. This includes the impact of globalisation and trade liberalisation on such regulation.^92^ Across the sample, emphasis is placed on tobacco,^26^ alcohol^25^ and unhealthy foods.^55,81^ The reviewed literature largely overlooks the gambling, arms, fossil fuel, social media and other industries which also harm health. There is reasonably little about the investment community or the legal, accountancy, and management consultant industry and the role they play in supporting and defending unhealthy societies.

 The “products” literature in our sample often consider the impact of the commercial determinants in relation to other components in our heuristic, ie, people,^4^ places,^25,73^ and planet.^19^ We have seen a growth of literature on commercial determinants in recent years.^13,93^ Lacy-Nichols and Marten^91^ illuminate how corporate power can keep commercial drivers off policy agendas and the perpetuation of societal investment in the downstream “illness industry.”

 The role of “powerful players”^73^ and their framing of lifestyles, personal responsibility and choice as presenting roadblocks to healthy societies represents a thread through the literature.^28,42,91^ Some frameworks encourage equity-oriented policies. For example, we saw calls for tobacco and alcohol policies to consider the unequal distribution of power and resources,^25,26,42^ by enforcing, for example, the prohibition of sales to minors in disadvantaged communities.^26^ Others call for support to civil society to apply pressure for government action to curb industry harms and influence on public policy.^28^ However, despite repeated calls to address CDoH, the public health responses to such drivers remain inadequate, including in the prevention of non-communicable diseases. Some argue this may be due to: (1) institutional inertia in governance processes, priorities and policies; (2) minimal civil society activism demanding political and policy responses; and (3) resistance to change from the commercial actors profiting from prevailing arrangements.^71^

 We found little treatment of healthy products, ensuring access to healthy diets, or devices and technology in ways that can be empowering and enhance well-being.^42,55^ Alongside this, who ought to be targeted to access subsidised healthy products was given very limited coverage.^81^ However, there was some attention to the need for sustainable food systems.^23,28,55^

 The role of the commercial sector in influencing political and scientific processes receives scant attention in our sampled documents. Lobbying by companies and their associations for favourable policy environments as well as their role in underpinning the political, economic and normative systems that enable these actors to operate are considered in more recent work.^91,94,95^

####  Planet

 The planet is the final component of our heuristic. While consideration of the natural environment on health is evident in some early documents,^3,15^ concepts such as One Health^24,40,56^ and planetary health^29,55^ are more recent. A unique feature of this literature is the call for urgent action, at all levels.^55^ Placed in a broader context, the planetary health literature seeks to expand understanding of what had until that point been the somewhat more technically confined concept of One Health – examination of health and disease occurrence in human and animal populations.^96,97^

 In line with the discussion on values above,^19^ documents in our sample challenge an over-reliance on gross domestic product as a measure of human progress, calling for the inclusion of indicators relating to human-caused health and environmental harms.^29^ Such calls for rebalancing of societal values are increasingly prominent.^19,40^ Some argue that planetary health is not a new concept or discipline, but rather has historical threads in integrative medicine, science of the microbiome and holistic medicine approaches.^97^ It is further argued that if analysts and advocates continue to perpetuate the neo-colonial capitalist world order that de-politicises and de-historicises the environment and the beings residing within it, equitable healthy societies will remain elusive.^98^

 Criticisms of the planetary health literature^29^ include that they continue to draw boundaries and attempt to exert control around natural phenomenon, reinforcing “a Western representation of our relationship to nature…founded on mastery” and techno-financial fixes located in the dominant neo-liberal paradigm.^99^ For example, while planetary health calls for training of indigenous community members to protect health and biodiversity, the possibility that learning, knowledge and capacity can flow in the opposite direction, ie, from indigenous peoples, is too often ignored.^100^ Proponents of alternative concepts like “One Health of Peripheries” argue that “Planetary Health and One Health can be read as proposals for preserving the capitalist order; more specifically, these two approaches arguably perpetuate the prevention and control of environmental deterioration and animal diseases to avoid more instability in the capitalist order. As might be expected, the colonial aspects of these proposals have not gone unnoticed.”^98^ Monbiot^101^ adds the additional concern that approaches to planetary health create silos (ie, climate change, deforestation) and depoliticise the challenge by focusing on individuals rather than economic and political systems to respond to planetary challenges.^99^ Recent contributions draw attention to the economic losses of climate change and the cost of inaction.^102-104^ These include food unavailability, food insecurity and income inequality,^102^ perpetuated by vested interests to “preserve the status quo”^103^ and run against healthy societies. More progressive approaches call for fundamental “reorientation of human systems,”^28^ while others critique the approach for its often myopic consideration of other planetary inhabitants — animals, plants — in instrumentalist terms (as vectors of disease transmission or as food).^97,102,103^

## Discussion

 The discourse on health in the health sector rarely concerns itself with health, but rather with illness, treating disease or strengthening healthcare systems. We sought to better understand what similar discussions about “health” rather than disease offer.

###  Not Another Framework

 The literature provides numerous frameworks to help guide analysis and inform action. These include Dahlgren and Whitehead’s seminal work on social determinants.^42^ The VicHealth’s Fair Foundation Framework builds on this.^35^ The structural violence framework takes the SDoH in more radical directions, drawing on political economy to focus on the upstream social and political systems that beget “causes of causes.”^37^ The framing of health inequities as the result of deliberate acts of violence as well as the far-reaching questions about how to achieve healthy societies set this framework apart from the work on social determinants.^37^ The 2019 Commission on the “global syndemic of obesity, undernutrition, and climate change” provides a different kind of framework presenting double- and triple-duty actions, simultaneously addressing multiple determinants driving the concurrent epidemics.^28^

 While these frameworks provide useful insights on some issues, they often overlook others. For example, one identifies policy levers to deliver SDG 3 (ensure healthy lives and promote well-being), yet it does so by focusing on universal health coverage neglecting considerations of societal drivers of health.^54^ These frameworks all have utility, yet as Givens et al^65^ note, the diversity and inconsistency of “inclusion of the potential categories or dimensions of drivers of health and equity” result in frameworks often failing to deliver “conceptual clarity on what shapes health and equity for the field of population health.”

 It is sobering to consider that the frameworks have failed to achieve their desired outcomes. For example, Alma Ata^2^ called for action on the underlying causes of ill health, yet “health for all” remains little more than a slogan. Is this a problem with the frameworks (for example that they are northern-centric and fail to consider a plurality of ideas^11^) or with the implementation of the frameworks? It was rare to find frameworks with the ambition to unify elements identified across literatures into one framework. This finding is echoed in a review of health and equity frameworks.^65^ As a result, the social determinants approach, for example, is considered inadequate and in need of expansion.^24,65,105^

 The majority of frameworks were created in an era dominated by neo-liberal ideology. Their failure to acknowledge, critique or offer alternatives to prevailing orthodoxy or consider the implications of neo-liberalism for the goals advocated, suggests the limited utility of the frameworks. It is equally tenable that there is no perfect framework that can address all the components and their political dimensions. This suggests that the place to start is from commitments already made and to understand their implications: what levers can be used and how those levers may be “activated” or enabled. We expand on this in our companion paper.^12^

###  Blinkered Boundary Thinking

 Another reflection on our sample relates to the interconnected nature of the elements (and concepts). Our heuristic presents a two-dimensional canvas onto which to map how inputs can be transformed through a set of values, components, and, as explored in our companion piece, policy levers and enablers.^12^ While these parts are often acted upon separately, these factors interact either exacerbating health inequities or enhancing the conditions which enable health and well-being.

 Drawing boundaries for the purposes of research and policy is understandable–putting boxes around social determinants, urban health, healthy diets, and so on enables us to make sense of complexity. Yet these categories are not, as argued by Kant and others more recently, the “thing in itself”– they are artifacts and fields created in our minds and actions.^101^ They reinforce silos and create artificial lenses obscuring complexity. In so doing, they fail to appreciate potential cascading benefits of interventions and/or fail to consider potential negative externalities. Overcoming this tendency to compartmentalise requires complexity-based approaches and systems thinking^20,53,86^ that consider the interconnections required to nurture and sustain healthy societies.

###  Privileged Positions and Paradigms

 Adding to the complex terrain are the lenses through which the challenges are analysed. The examined literature focuses primarily on high-income settings. There is a particular gap in documents that examined environments occupied by the urban poor—something that is increasingly recognised in some of the CDoH literature.^106,107^ The lack of LMIC perspectives (See Supplementary file 1) suggests these frameworks may not address the issues in ways meaningful to the majority of the world’s population.^11^ However, even within LMICs, due in large part to the political economy and history of public health — which has placed a great deal of emphasis on population control in LMICs — the health sector has been largely preoccupied with maternal and child health limiting broader considerations.

 The literature also reflects the dominant influence of biomedical conceptualisations of health and illness, a feature also concluded by Loewenson et al in their regional review of healthy societies.^11^ Yet, given the biomedical orientation within much of the health sector, how can leaders within the sector promote healthy societies approaches across other sectors that do not fall back on bio-medical solutions? Acknowledging and addressing this challenge appears to be critical to advancing the healthy societies agenda.

###  What Is Valued in a Healthy Society?

 Our analysis highlighted the integral role of values to the creation of healthy societies. A healthy societies paradigm promotes alternative endpoints to development, eschewing short-term economic growth in favour of health, equity and well-being. It also embraces a set of supportive values to guide investment, research and action. However, like Givens and colleagues,^65^ this review identified a lack of attention to the strategic actions required to shift societal values. For example, while equity was the value identified in the greatest number of documents, few frameworks were explicit about the underlying theories of inequity, the neo-liberal values which sustain them, or actions to redress inequities.^41,44^ More recent documents encourage consideration of not merely equity in the distribution of outcomes but the costs associated with prioritising specific social values over others. For example, the social costs of prioritising economic outcomes over health and well-being focused outcomes.^19^ Encouragingly, the planetary health frameworks increasingly call for reconsideration of core societal values.^19,29^

 In many of the government documents, the presentation of value statements in the preambulatory sections placed relatively less emphasis on what equity should look like, particularly in terms of institutional, community or system arrangements and processes.^30,41^ Despite sustained calls for the privileging of a set of core values for healthy societies — calls reinforced by the COVID-19 pandemic — societies continue to prioritize a competing set of values (eg, economic growth or efficiency) which often undermine public health given the nature of growth and the distribution thereof.^19^ Future research should monitor the outcomes associated with value-based actions and their impact on influencing health-facilitating societal norms.

 Several values arguably crucial to healthy societies were not addressed in our sample. This includes contestation and resistance. If we are to change the systems that perpetuate poor health, we need to allow/create spaces for voices that challenge prevailing interests. A study of intersectoral collaboration around deforestation argued that where collaboration does not work, contestation does in relation to environmental justice.^108^ The history of the AIDS movement reveals that resistance and contestation was critical to reverse the criminalisation of gay sex, drug use and sex work and enable effective HIV prevention measures.^109,110^ The healthy societies literature has yet to take such a subversive turn.

###  Capitalism and Neo-Liberalism: Rate Limiting Factors to the Attainment of Healthy Societies

 All four components are mediated by modern capitalism, even if this is underplayed in the literature, except for some focused on products and planet. healthy societies are premised on the ability of people to enjoy a healthy work/life balance, engage in decent and reasonably remunerated work and enjoy access to health promoting environments (places), and to nutritious food (products) that is sustainably produced (planet). To elaborate in relation to food, it is increasingly evident that foods need to be judged not only on their impact on health, but on the environmental, social and economic conditions of their production, processing and distribution^55^ — hence the growth of front of pack labelling and Environmental, Social, and Governance investing initiatives. All these interactions are influenced by the structures and practices of capitalism.^111^

 The imprint of capitalism, and its neo-liberal globalised variant, and the values of extraction, growth, accumulation and concentration, resulting in the rolling back of the state, regulatory apparatus and social safety nets can be felt across all four components of the framework — as COVID-19 revealed. Yet the literature is largely silent on these systemic drivers of ill-health — except to some extent questing the prevailing narrative of the agency of the individual to protect and promote their own health in a deeply unequal world.

###  Limitations

 Given the aims of this research and the volume of associated literature (one review, for example, found 36 frameworks concerning social determinants), select documents were identified to capture key conceptual frameworks. We recognise the limitations of the methodology in relation to both potentially omitting important sources as well as biases inherent in including others given that it would be very difficult to systematically identify every potentially relevant document through a close-ended search. Moreover, given the nature of much the source material, we did not appraise the validity, reliability or quality of concepts proposed in frameworks and literature, but rather reflected on their contribution to understanding healthy societies.

 As a team of researchers from countries in the Global North and South, our process was iterative and recursive, to build, question and rebuild our analytical framework. It would have been influenced by own positionality. All authors are social scientists with strong orientations towards equity, human rights and social justice. The analysis was limited to English-language documents, which may play some role in why we have a preponderance of documents from high-income, anglophone countries. We recognize that this creates a bias in terms of perspectives represented in our analysis. This non-exhaustive research process is limited by our global approach, with limited analysis of region-specific perspectives.

## Conclusion

 Our paper provides an overview of the “what” of healthy societies. Calls for what Vinuales and colleagues recently refer to as “deep prevention” (ie, structural and systemic reforms)^112^ have been long standing but have gone largely unheeded. Rather than offer a diagnosis of why past efforts have not been successful, most documents envision and re-envision ever more components and connections. The omission of introspection reflects our finding that literature is apolitical and/or fails to grapple with the question of whose interests are at likely to be affected if the frameworks were to be implemented. Future work should seek to identify what did not work in the past while seeking to identify strategies most effective in addressing likely political and ideological opposition.

 The literature provides an alternative vision of sustainable development for healthy societies — including an alternative set of values and approaches to address structural drivers of health inequities. Yet these values have failed to take root in mainstream society while the vision to transform approaches focusing on people, places, products and planet has largely been ignored. This might be explained by the vastness of the terrain and the complexity inherent in comprehensive upstream responses. It might also be a function of the failure of the frameworks to provide convincing narratives or to consider how to engage with or confront vested interests.^113^

 In their pandemic responses, most states vastly increased investments in social protection (for example, paying wages for furloughed workers). This suggests what is possible with political resolve. Recent initiatives such as the meetings on healthy societies for Healthy Populations, convened by the Government of Sweden, WHO, the Alliance for Health Policy and Systems Research, Wilton Park, and the Wellcome Trust from 2020-2022^114^ provide grounds for optimism.^115^ But what is likely ultimately needed is for social movements to get behind the healthy societies agenda.

 Future study and action would be enhanced by taking more expansive views of the terrain, exploring the intersections and interstices, and applying a more critical lens to what accounts for the limited progress on this agenda to date.

 The COVID-19 pandemic revealed decades of underinvestment in public health that left societies vulnerable and lacking resilience.^116^ The twin pandemics of COVID-19 and inequality focussed unprecedented attention on the need and opportunities^117^ to address structural, social, economic and political drivers of health inequalities. The UN’s research roadmap for recovery argues that the pandemic “brought into sharp focus the need for ambitious plans that reimagine and rebuild health, social and economic systems so that they leave no one behind” while providing an “historic opportunity to reimagine societies using a human rights lens.”^40^ We agree. And while many of the healthy societies frameworks point in those directions, what remains is mobilising the grass roots politically to create demands to move from aspirations to attainment of health and well-being for all.

## Acknowledgements

 The authors acknowledge Sreejini Jaya for initial data extraction and coding.

## Ethical issues

 Not applicable.

## Competing interests

 Authors declare that they have no competing interests.

## Disclaimer

 Robert Marten and Sonam Yangchen are staff members of the Alliance for Health Policy and Systems Research, a WHO-hosted partnership.

## Funding

 This research received funding from The Alliance for Health Policy and Systems Research.

## Supplementary files


Supplementary file 1. Sample Documents.

Supplementary file 2. Data Extraction Template.

Supplementary file 3. PRISMA-ScR Flow Diagram.

Supplementary file 4. Values Present in the Sample Documents.

## Endnotes

 [1] The concept of the black box derives from Easton’s model of the political system through which “inputs” (demands, resources and support) are transformed into “outputs” (public policies and public goods) in what is perceived as an opaque box of policy-making.^18^
