# Supplementary material for: What Are Healthy Societies? A Thematic Analysis of Relevant Conceptual Frameworks
Source: Int J Health Policy Manag. 2023 Nov 7;12:7450. doi: 10.34172/ijhpm.2023.7450 (PMC10699824; doi:10.34172/ijhpm.2023.7450)
Supplement: Supplementary file 1 — Sample Documents. [file ijhpm-12-7450-s001.pdf]

Article title: What Are Healthy Societies? A Thematic Analysis of Relevant Conceptual Frameworks

Journal name: International Journal of Health Policy and Management (IJHPM)

Authors' information: Kent Buse[1]*, Amy Bestman[2], Siddharth Srivastava[3], Robert Marten[4], Sonam Yangchen[4], Devaki Nambiar[3,2,5]

[1]The George Institute for Global Health, Imperial College London, London, UK.

[2]Faculty of Medicine, University of New South Wales, Sydney, NSW, Australia.

[3]The George Institute for Global Health, New Delhi, India.

[4]The Alliance for Health Policy and Systems Research, World Health Organization (WHO), Geneva, Switzerland.

[5]Prasanna School of Public Health, Manipal Academy of Higher Education, Manipal, India.

***Correspondence to:** Kent Buse; Email: kentbuse@gmail.com

Citation: Buse K, Bestman A, Srivastava S, Marten R, Yangchen S, Nambiar D. What are healthy societies? A thematic analysis of relevant conceptual frameworks. Int J Health Policy Manag. 2023;12:7450. doi:10.34172/ijhpm.2023.7450

Supplementary file 1. Sample Documents

Overview of included documents

Year	Author	Authorship	Framework/ document title	Source	Type of document	Funding source	Purpose (overarching aim)	Components	Levers & enablers	Levels	Res. Ag
**1974**	Lalonde [15]	HIC	A new perspective on the health of Canadians: A working document.	Google scholar	Working document	Government of Canada	This report states its purpose is to "to show the links between different kinds of mortality and illness on the one hand and their underlying causes on the other. Only when these links are known will it be possible to make judgments on whether certain risks are worth taking or certain sacrifices are worth making."	People, places, planet	RFM<br>IA<br>RS	Country, community	✓
**1978**	WHO[2]	INT	Primary health care: report of the International Conference on primary health care, Alma-Ata	Van Olmen et al.	Conference declaration	World Health Organization (WHO) and the United Nations Children's Fund	This declaration from the International Conference on Primary Health Care (1978) calls for "the need for urgent action by all governments, all health and development workers, and the world community to protect and promote the health of all the people of the world."	People	RFM<br>PWA<br>PM	Global, country	✓
**1986**	WHO Euro[3]	INT	Ottawa Charter for Health Promotion	Van Olmen et al.	Conference charter	WHO	The conference was the first International Conference on Health Promotion (held in Ottawa, Canada in 1986). This charter for Health Promotion identified six priority	People, places	RFM<br>IA<br>RS	Global, community	✓

Year	Author	Authorship	Framework/ document title	Source	Type of document	Funding source	Purpose (overarching aim)	Components	Levers & enablers	Levels	Res. Ag
							action areas to achieve Health for All: 1) build healthy public policy, 2) create supportive environments, 3) strengthen community actions, 4) develop personal skills, 5) reorient health services and 6) considerations moving into the future.		PWA PM		
**1993**	Sen[68]	HIC	Capability and Well-Being	Added by authors	Book chapter	Not declared	This chapter provides an overview to Sen's Capability framework (representative of the alternative combinations of 'functionings' a person can achieve).	People	PM	N/A	X
**1996**	Hamilton & Bhatti[38]	HIC	Population health promotion: An integrated model of population health and health promotion.	Google scholar	Government working document	Government of Canada	This paper combines ideas relating to health promotion and population health to develop an integrated Population Health Promotion Model.	People	GK RS	N/A	✓
**2000**	Berkman et al.[74]	HIC	From social integration to health: Durkheim in the new millennium	Google scholar	Review	Not declared	This paper presents a conceptual model that encompasses social networks, social ties and social integration in a single framework.	People	GK	N/A	X
**2000**	People 's Health Movement[46]	INT	The People's Charter for Health	Added by authors	Conference charter	Not declared	In response to lack of action on Alma Ata calls, this charter was developed at the "People's Health Assembly" in Bangladesh (2000). The charter states that health and well-being are a fundamental human right and calls for "universal access to quality health care, education and other social services according to people's needs"	People	RFM IA PWA PM KG	Country Global Community	X
**2001**	Participants of the conference on "Health Care for All"[39]	INT	Declaration on 'health care for all'	Van Olmen et al.	Conference declaration	Not declared	This declaration, formulated by a ministerial working group and endorsed by the participants of the conference on "Health Care for All" (2001), calls on national governments, international organisations, all agencies and individuals concerned with health and development to support health initiatives and commitments to "Health Care for All".	People	RFM IA PWA GK	Country, global	✓
**2003**	Chen & Narasimhan[80]	HIC	Human security and global health	Added by authors	Journal article	Not stated	The paper "considers the concept of human security expands understanding of the links between health and human development, the ways in which human security is linked to global health, particularly as regards violence and conflict, global infectious diseases, and poverty and inequality. The authors then draw out the implications for policymaking."	People	RS PWA RS	Global	X

Year	Author	Authorship	Framework/ document title	Source	Type of document	Funding source	Purpose (overarching aim)	Components	Levers & enablers	Levels	Res. Ag
**2003**	Ogata & Sen[60]	HIC	Human Security Now: Commission on Human Security	Added by authors	Commission Report	Ministry of Foreign Affairs of Japan, Government of Sweden, the World Bank, the Rockefeller Foundation, and the Japan Center for International Exchange.	This report from the Commission on Human Security focuses on areas such as conflict and poverty, protecting people during violent conflict overcoming economic insecurities and guaranteeing the availability and affordability of essential health care. Recommendations include "policies aimed at both empowerment and protection, and focus on what can be done in the short and the long run to enhance the opportunities for eliminating insecurities across the world."	People	RFM IA RS PM GK	Country, global	✓
**2004**	Schulz & Northridge[49]	HIC	Social determinants of health: implications for environmental health promotion	Google scholar	Journal article	Not declared	In this paper, authors seek to further understand the "mechanisms through which social factors contribute to disparate environmental exposures and health inequalities." This work proposed a conceptual framework for environmental health promotion.	People, places	IA RS PWA	Community, country	✓
**2005**	Gasper	HIC	Securing humanity: situating 'human security' as concept and discourse	Added by authors	Journal article	Not declared	This paper "maps the increasingly complex and sometimes confused field of concepts and usages to help to place 'human security' in meaningful context, and to clarify how it relates to 'human development'."	People	PWA	Community, country, region, global	X
**2006**	Barton & Grant[50]	HIC	A health map for the local human habitat	Maani et al.	Editorial	Not declared	This editorial presents the health map, "a tool to improve understanding and foster collaboration between planning and health decision-makers."	Places, people, planet	IA	Community, country, global	✓
**2006**	Dahlgren & Whitehead[42]	HIC	European strategies for tackling social inequities in health: Levelling up Part 2	Maani et al.	WHO report	WHO	This document aims to support "policy-makers in their efforts to address social inequities in health in a Europe that is rapidly changing."	People, places, products	RFM IA RS PM GK RS	Region, country, community, global	✓
**2006**	Etches et al.	HIC	Measuring population health: a review of indicators	Google scholar	Journal article	Not declared	This paper proposes "a conceptual framework for using indicators to report on all the domains of population health."	People	RS GK	N/A	✓

Year	Author	Authorship	Framework/ document title	Source	Type of document	Funding source	Purpose (overarching aim)	Components	Levers & enablers	Levels	Res. Ag
**2006**	Ståhl et al.[52]	HIC	Health in all policies: prospects and potentials	Google scholar	Project report	European Union Public Health Programme	This report, published by the Finnish Ministry of Social Affairs and Health (under the auspices of the European Observatory on Health Systems and Policies), has been divided into five parts: 1) theories, concepts and challenges in regard to HiAP, 2) concrete examples of how HiAP has been implemented, 3) Governance, 4) Health impact assessment and 5) Conclusions and the way forward.	People, places, products	RFM IA PWA PM GK	Country, region, global, community	✓
**2006**	Whitehead & Dahlgren[4] [4]	HIC	Concepts and principles for tackling social inequities in health: Levelling up Part 1	Maani et al.	WHO report	WHO	This report notes that "to be effective in tackling social inequities in health, policy-makers and practitioners need a sound understanding of the current evidence about the key determinants and ways in which health systems can confront them in different country contexts." This report therefore aims to "help policy-makers in their efforts to address social inequities".	People, places, products	PWA PM GK	Region, country, community, global	✓
**2007**	Siddiq et al.[45]	Not disclosed	Total environment assessment model for early child development: evidence report for the World Health Organization's Commission on the social determinants of health	Google scholar	Evidence report	WHO	This report for the for WHOs Commission on the Social Determinants of Health aims to "1) demonstrate which environments matter most for children, 2) review which environmental configurations are optimal for early childhood development (ECD), 3) determine the "contingency relationships" that connect the broader socioeconomic context of society to the quality of nurturing in intimate environments and, 4) highlight opportunities to foster nurturant conditions for children at multiple levels of society and by multiple means."	People, places	RFM IA PM GK	Community, country, region, global	✓
**2007**	Whitehead[61]	HIC	A typology of actions to tackle social inequalities in health.	Google scholar	Editorial	Not declared	This paper presents a typology of the most prominent types of actions to address health inequalities to "broaden the understanding of the range of different interventions available and their potential effectiveness for the task in hand, and to avoid the tendency to focus on one type of intervention neglecting the others."	People	IA GK	Community, country	X
**2008**	Commission on Social Determinants of Health.[4]	INT	Closing the gap in a generation: health equity through action on the social determinants of health: final report of the commission on social determinants of health	Van Olmen et al.	Comission report	WHO	This report contains the "material for developing solutions to the gross inequities between and within countries" for "global action on the social determinants of health with the aim of achieving health equity."	People, places, products, planet	RFM IA RS PWA PM GK	Country, region, global, community	✓

Year	Author	Authorship	Framework/ document title	Source	Type of document	Funding source	Purpose (overarching aim)	Components	Levers & enablers	Levels	Res. Ag
**2008**	Dyck[78]	HIC	Social determinants of Métis health. Canada: Métis Centre	Google scholar	Report	National Aboriginal Health Organization (Canada)	This report explores the social determinants of health within the context of Métis (Aboriginal people) health priorities. This report presents frameworks for Métis health determinants framework and the determinants of Métis well-being.	People, places, planet	RS PM GK	Community, country	✓
**2008**	Hiatt & Breen[43]	HIC	The social determinants of cancer: a challenge for transdisciplinary science	Google scholar	Journal article	Not declared	This paper presents a “conceptual framework is designed to encourage transdisciplinary research that will integrate social determinants into cancer research.”	People	IA GK	N/A	✓
**2008**	Kawachi et al.[69]	HIC	Social Capital and Health: A Decade of Progress and Beyond	Added by authors	Book chapter	Not declared	This chapter defines social capital and related concepts such as bonding and bridging social capital. The chapter also briefly examines social capital and health research.	People	PM GK	N/A	✓
**2009**	Fox & Meier[64]	HIC	Health as freedom: addressing social determinants of global health inequities through the human right to development	Google scholar	Journal article	Not declared	Article proposes that states codify, in international human rights law, an ethical framework for ameliorating social determinants of health, providing an institutional mechanism for developing states to realize global health and social justice goals through the human right to development.	People	RFM PWA	Country, region	X
**2010**	Bambra et al.[89]	HIC	Tackling the wider social determinants of health and health inequalities: evidence from systematic reviews	Google scholar	Journal article	The work was supported by the Public Health Research Consortium (who are funded by the English Department of Health Policy Research Programme).	This systematic review synthesises “systematic reviews on the effects on health and health inequalities of interventions aimed at influencing the social determinants of health.”	People, places	IA GK	Country	✓
**2010**	Bozorgmehr[21]	HIC	Rethinking the 'global' in global health: a dialectic approach	Google scholar	Journal article	Article processing charge was covered by University Medical	This discussion paper aims to provide a way forward towards an understanding 'global health' while avoiding redundancy. Further attention is payed to normative objectives attached to 'global health' definitions and to paradoxes involved in attempts to define the field. The paper presents two frameworks, the concept of global	Not specified	RFM PWA GK	Global	✓

Year	Author	Authorship	Framework/ document title	Source	Type of document	Funding source	Purpose (overarching aim)	Components	Levers & enablers	Levels	Res. Ag
						Center Berlin, Germany	health (including the territorial dimension) and Supraterritorial links between the Social Determinants of Maternal Mortality.				
**2010**	Solar & Irwin[59]	Int	A conceptual framework for action on the social determinants of health	Maani et al.	WHO discussion paper	WHO	This discussion paper describes conceptual frameworks for science and policy for health equity	People, places, products	RFM IA RS PWA PM GK	Country, region, global, community	✓
**2011**	Braveman et al.[48]	HIC	The social determinants of health: coming of age	Google scholar	Journal article	Not declared	This paper reviews current knowledge about the "health effects of social (including economic) factors, knowledge gaps, and research priorities" focusing on upstream social determinants-including economic resources, education, and racial discrimination -that fundamentally shape the downstream determinants, such as behaviours, targeted by most interventions.	People	IA PWA PM	Country	✓
**2012**	Golden & Earp[51]	HIC	Social ecological approaches to individuals and their contexts: twenty years of health education & behavior health promotion interventions	Added by authors	Journal article	No financial support for research, authorship, and/or publication	This review paper developed a coding system "to identify the ecological levels that health promotion programs target". Author then examined "which ecological levels received the most attention in intervention efforts and determine the extent to which programs simultaneously tar-get multiple levels of change."	Places, people	GK	Country, community	✓
**2012**	Lorenc et al.[84]	HIC	Crime, fear of crime, environment, and mental health and wellbeing: mapping review of theories and causal pathways	Added by authors	Journal article	Funded by the National Institute of Health Research	This review paper aimed to "present a holistic framework tracing the theoretical links between crime, fear of crime, the environment and health and wellbeing" and to use the framework to as an example of one way to construct "theory in the context of undertaking evidence synthesis of the effectiveness of complex social interventions."	Places, people	GK	N/A	✓
**2013**	Board on Population Health Public Health Practice et al.[87]	HIC	US health in international perspective: Shorter lives, poorer health	Google scholar	Book	National Institutes of Health, National Research Council and the Institute of Medicine	This report addressed three aims, "1) to document the nature and scope of the U.S. health disadvantage, 2) to explore potential explanations for this disadvantage, and 3) to propose next steps for the field."	People, places, products	RS PM GK	Country	✓

Year	Author	Authorship	Framework/ document title	Source	Type of document	Funding source	Purpose (overarching aim)	Components	Levers & enablers	Levels	Res. Ag
**2013**	WHO Regional Committee for Europe[20]	Int	Health 2020: a European policy framework and strategy for the 21st century	Google scholar	WHO policy framework	WHO	This document provides "a flexible framework for policy and practice across [WHO] member States of the European Region, building on a long history of global and regional policy thinking."	People, places, products, planet	RFM IA RS PWA PM GK	Region, country	✓
**2014**	Krumeich & Meershoek[106]	HIC	Health in global context; beyond the social determinants of health?	Google scholar	Journal article	Not declared	This review article aims to "explore whether and how social determinants of health frameworks can be translated to effectively inform particular national health policies."	People, places	GK	Global, country, community	✓
**2014**	Welsh et al.[75]	HIC	Evidence Review: Addressing the social determinants of inequities in mental wellbeing of children and adolescents	Google scholar	Evidence review	VicHealth	This evidence review of Australian and international literature aimed to "provide an overview of the social determinants of inequities in mental wellbeing in children and adolescents, to provide evidence on interventions which address inequities in wellbeing and to identify the evidence and conceptual gaps."	People	IA RS GK	N/A	✓
**2014**	WHO[62]	INT	Health in all policies: Helsinki statement. Framework for country action.	Maani et al.	Conference statement and framework	Conference organised by WHO and Ministry of Social Affairs and Health (Finland)	The Helsinki Statement on Health in All Policies was endorsed by attendees at the 8th Global Conference on Health Promotion 2013. The 'Framework for Country Action' provides practical guidance to apply Health in All Policies (HiAP) approach in decision making and implementation at national and subnational levels.	People	RFM IA PWA PM GK	Country	✓
**2015**	Ball et al.[82]	HIC	Evidence review: addressing the social determinants of inequities in physical activity and related health outcomes	Google scholar	Evidence review	VicHealth	This report aims to "summarise the nature and quality of the evidence on social determinants of inequities in physical activity, with a view to identifying promising approaches to promoting equity in physical activity and related health outcomes at each layer of the Fair Foundations: The VicHealth framework for health equity."	People, places	RFM IA GK	Community	✓
**2015**	Friel et al.[81]	HIC	Evidence review: Addressing the social determinants of inequities in healthy eating	Google scholar	Evidence review	VicHealth	This evidence review of Australian and international literature aims to "identify promising evidence-based approaches for promoting equity in healthy eating at each level of the Fair Foundations Framework."	Products, people, places	RFM IA PWA GK	Community	✓
**2015**	Newman et al.[73]	HIC	Evidence Review: Settings for Addressing	Google scholar	Evidence review	VicHealth	This evidence review aims to identify the extent to which settings address the social determinants of health inequities	People, places	RFM IA	Community	✓

Year	Author	Authorship	Framework/ document title	Source	Type of document	Funding source	Purpose (overarching aim)	Components	Levers & enablers	Levels	Res. Ag
			the Social Determinants of Health Inequities						PM GK		
**2015**	Purcell [26]	HIC	Evidence review: Addressing the social determinants of inequities in tobacco use	Google scholar	Evidence review	VicHealth	This evidence review aimed to "synthesise current Australian and international evidence describing the social determinants of inequities in relation to tobacco use, identify promising strategies which could feasibly be implemented, and identify key gaps in the evidence base and make recommendations for future research priorities in relation to tobacco use and inequity."	Products, people, places	RFM IA PWA GK	Community, country	✓
**2015**	VicHealth. [25]	HIC	Evidence review: the social determinants of inequities in alcohol consumption and alcohol-related health outcomes	Google scholar	Evidence review	VicHealth	This evidence review aims to "provide policy makers and practitioners in Victoria and across Australia with practical, evidence-based guidance on promoting equity in the reduction of alcohol-related harms."	Products, people, places	RFM RS PWA PM GK	Community, country	✓
**2015**	VicHealth [35]	HIC	Fair Foundations: the VicHealth Framework for Health Equity	Google scholar	Conceptual framework	VicHealth	This planning tool, based on a conceptual framework developed by the World Health Organization Commission on the Social Determinants of Health, aims to "increase understanding of the social determinants of health inequities and suggests entry points for action, for the development of policies and programs that promote health equity."	People, places,	RFM IA PWA PM GK	Community, country	X
**2015**	VicHealth [85]	HIC	Promoting equity in early childhood development for health equity through the life course	Google scholar	Evidence review	VicHealth	This evidence summary aims to provide "provides policy makers and practitioners in Victoria and across Australia with practical, evidence-based guidance on promoting more equitable outcomes in child health and development."	People, places	RFM IA PM GK	Community, country	✓
**2015**	VicHealth [86]	HIC	Promoting equity through social innovation	Google scholar	Evidence review	VicHealth	This evidence summary aims to provide "policy makers and practitioners in Victoria and across Australia with practical, evidence-based guidance on using social innovation to promote health equity."	People, places	IA PM GK	Community, country	✓
**2015**	Whitmee et al.[29]	HIC + LMIC	Safeguarding human health in the Anthropocene epoch: report of The Rockefeller Foundation–Lancet Commission on planetary health.	Added by authors	Commission report	The Rockefeller Foundation; Medical Research Council and from the Wellcome Trust;	This report explores 4 key areas: "1) natural systems are being degraded to an extent unprecedented in human history", 2) "societies face clear and potent dangers that require urgent and transformative actions to protect present and future generations", 3) "the present systems of governance and organisation of human knowledge are inadequate to address the threats to planetary health", and 4) "solutions should be based on the redefinition of prosperity to focus on the enhancement of quality of life	Planet, people, places, products	RFM IA RS PWA PM GK	Global, region	✓

Year	Author	Authorship	Framework/ document title	Source	Type of document	Funding source	Purpose (overarching aim)	Components	Levers & enablers	Levels	Res. Ag
						Natural Environment Research Council; B&MGF; Winslow Foundation	and delivery of improved health for all, together with respect for the integrity of natural systems."				
**2016**	Attendees of the 9th Global Conference on Health Promotion [58]	INT	Shanghai Declaration on promoting health in the 2030 Agenda for Sustainable Development	Added by authors	Conference declaration	Conference jointly organized by the Government of China and WHO	This conference declaration, endorsed by participants at the 9th Global Conference on Health Promotion (Shanghai, 2016), contains statements and commitments to action related to achieving the United Nations Development Agenda 2030 and its Sustainable Development Goals.	People, places	RFM IA PM	Global	X
**2016**	Graham & White[24]	HIC	Social determinants and lifestyles: integrating environmental and public health perspectives	Google scholar	Journal article	Draws on research funded by the Economic and Social Research Council University of York ESRC Impact Accelerator award.	This integrative review focuses on "social determinants and lifestyles as two 'bridging' concepts between the fields of public health and environmental sustainability", drawing on "established frameworks to consider the position of the natural environment within social determinants of health frameworks and the position of social determinants within environmental frameworks."	People, places, planet, products	IA PM GK	N/A	✓
**2017**	Buse et al.[57]	HIC	Healthy people and healthy profits? Elaborating a conceptual framework for governing the commercial determinants of non-communicable diseases and identifying options for reducing risk exposure.	Maani et al.	Journal article	Small grants scheme operated by UCL Global Governance Institute	This paper uses a conceptual framework to "review three models of governance of NCD risk: self-regulation by industry; hybrid models of public-private engagement; and public sector regulation" analysing "the challenges inherent in each model, and what is known (or not) about their impact on NCD outcomes."	Products, people	RFM IA PWA PM	Global, country	X

Year	Author	Authorship	Framework/ document title	Source	Type of document	Funding source	Purpose (overarching aim)	Components	Levers & enablers	Levels	Res. Ag
**2017**	Boswell et al.[76]	HIC	Keeping Us Well: How Non-health Charities Address the Social Determinants of Health	Maani et al.	Report	New Philanthropy Capital	This report aims to "support non-health charities to better understand and use the evidence about the social factors that impact on people's health and well-being."	People, places, planet	IA	Community, country	X
**2017**	de Leeuw[63]	HIC	Engagement of Sectors Other than Health in Integrated Health Governance, Policy, and Action	Added by authors	Journal article	None	This paper reviews 'conceptual foundations for integral health governance, policy, and action, delineates the different sectors and their possible engagement, and provides an overview of a continuum of methods of engagement with other sectors to secure integration'.	N/A	RFM IA PM GK	N/A	✓
**2017**	McNamara[92]	HIC	Trade liberalization and social determinants of health: a state of the literature review	Google scholar	Journal article	Not declared	This systematic review "provides a more complete picture of the pathways between trade liberalization and health, with special attention to the social determinants of health pathways."	N/A	RFM GK	Global	✓
**2018**	Koehler et al.[53]	HIC	Building healthy community environments: a public health approach	Google scholar	Journal article	Bloomberg American Health Initiative, Air Climate & Energy (ACE) Center Grant funded by US Environmental Protection Agency Assistance Agreement	This paper presents a conceptual framework that "that represents a shift from compartmentalized solutions toward an inclusive systems approach that encourages partnership across disciplines and sectors." The paper aims to provide "a guide for community leaders to consider the public health effects of decisions about the built environment."	Places	IA GK RS	Community	
**2018**	Kondo et al.[88]	HIC	Urban green space and its impact on human health	Added by authors	Journal article	National Institutes of Health grants and Centers for Disease Control and Prevention grant.	This paper aimed "to systematically review studies evaluating the association between urban green space and human health."	Places, people	GK	Community, country	✓

Year	Author	Authorship	Framework/ document title	Source	Type of document	Funding source	Purpose (overarching aim)	Components	Levers & enablers	Levels	Res. Ag
**2018**	WHO Independent High-level Commission on NCDs[23]	INT	Report of the Technical Consultation (21-22 March 2018)	Maani et al.	WHO report	WHO	This report proposes "practical recommendations to ensure that NCD targets are met, set within a framework to illustrate the interlinkages between recommendations," building upon the "commitments made by Heads of State and Government at the UN General Assembly in 2011, 2014 and 2015."	Products, people	RFM IA PWA PM GK	Global, regional, country, community	✓
**2018**	Vik & Carlquist[27]	HIC	Measuring subjective well-being for policy purposes: The example of well-being indicators in the WHO "Health 2020" framework	Added by authors	Journal article	University of Oslo	This paper "discusses the rationale for measuring national well-being and examines the use of subjectively oriented well-being measures in the context of public policy."	People	GK RS	Global	✓
**2019**	Cerf[54]	LMIC	Sustainable Development Goal Integration, Interdependence, and Implementation: the Environment–Economic–Health Nexus and Universal Health Coverage	Google scholar	Journal article	Not declared	This paper presents a general framework "for the implementation of the sustainable development goals (SDGs) with a focus on SDG 3 and one of its targets, universal health coverage (UHC). The robustly aligned environment–economic–health nexus is a key determinant for the successful implementation of UHC (and the SDGs)."	Places, people	IA PWA	Global, regional, country, community	X
**2019**	Government of New Zealand[30]	HIC	The Wellbeing Budget	Added by authors	Government document	New Zealand Treasury	The Minister of Finance describes the Wellbeing budget as a "significant departure from the status quo. Budgets have traditionally focused on a limited set of economic data. Success has been declared on the basis of a narrow range of indicators, like GDP growth. But New Zealanders have questioned that claim of success when they have seen other things that we hold dear – child wellbeing, a warm, dry home, or being able to swim in our rivers and lakes – getting steadily worse. The old ways have left too many people behind. It is time to change."	People, planet, places	RFM IA RS PWA PM	Country, community	
**2019**	Swinburn et al.[28]	HIC + LMIC	The global Syndemic of obesity, undernutrition, and climate change: the Lancet Commission report	Added by authors	Commission report	Redstone Global Center for Prevention and Wellness, George Washington	Authors describe the "three pandemics-obesity, undernutrition, and climate change-represent The Global Syndemic that affects most people in every country and region worldwide". The "Commission recommends comprehensive actions to address obesity within the context of The Global Syndemic, which represents the paramount health challenge for humans, the environment, and our planet in the 21st century."	Planet, products, people	RFM IA PWA PM GK	Global	✓

Year	Author	Authorship	Framework/ document title	Source	Type of document	Funding source	Purpose (overarching aim)	Components	Levers & enablers	Levels	Res. Ag
						University; Faculty of Medical and Health Sciences, University of Auckland; the Morgan Foundation, Wellington; the Warehouse Foundation, Auckland; Wellcome Trust, UK and the Science and Engineering Research Board, Department of Science and Technology, Government of India					
**2019**	Verma[31]	HIC	The Eight Manifestations of GNH: Multiple Meanings of a Development Alternative	Added by authors	Journal article	Not declared	This paper seeks to "respond to the dilemmas and challenges of understanding facing GNH by exploring its epistemological and historical foundations, and disentangling multiple meanings manifested in eight different forms" and contribute to "greater clarity to a growing body of multifarious writings on the subject, and more specifically, to an emerging body of scholarly literature on GNH by shedding explanatory light to the way it is conceptualized, operationalized, practiced, understood, internalized, and continuously undergoing change as it is refined and deepened over time."	People, planet	RS	Country	X

Year	Author	Authorship	Framework/ document title	Source	Type of document	Funding source	Purpose (overarching aim)	Components	Levers & enablers	Levels	Res. Ag
**2019**	WHO[10]	INT	Thirteenth General Programme of Work 2019–2023	Added by authors	General program of work	WHO	"GPW 13 sets out WHO's strategic direction, outlines how the Organization will proceed with its implementation and provides a framework to measure progress in this effort. GPW 13 will guide for each biennium stepwise progress in strategic priorities, the development of implementation plans, the programme budget, results frameworks and operational plans."	People, places, products, planet	RFM IA RS RFM PM GK	Global	✓
**2019**	Willett et al.[55]	HIC + LMIC	Food in the Anthropocene: the EAT–Lancet Commission on healthy diets from sustainable food systems	Added by authors	Lancet Commission report	The Wellcome Trust Children's Investment Fund Foundation	"This Commission brings together 19 Commissioners and 18 co-authors from 16 counties in various fields of human health, agriculture, political sciences, and environmental sustainability to develop global scientific targets based on the best evidence available for healthy diets and sustainable food production."	Planet, product, people	RFM IA PM GK	N/A	✓
**2020**	Amuasi et al.[56]	HIC + LMIC	Reconnecting for our future: the Lancet One Health commission	Added by authors	Commission report	German Federal Ministry of Education and Research and the Life Science Programme at the University of Oslo.	The objective of the Comission was to "synthesise the evidence supporting a One Health approach to enhancing health within an environment shared by humans and animals."	Planet, place, people	IA GK	Global	✓
**2020**	Hawkes & Buse[47]	HIC	The Politics of Gender and Global Health	Added by authors	Book chapter	Not declared	This chapter "presents a conceptual framework that explains the ways through which gender impacts [health] outcomes, namely how gender serves as and interacts with other determinants of health, how gender influences the differences in health-harming and health-affirming behaviours between men and women, and how gender impacts health programmes and delivery."	People	PWA PM GK	N/A	✓
**2020**	Herrick & Bell[37]	HIC	Concepts, disciplines and politics: on 'structural violence' and the 'social determinants of health	Added by authors	Journal article	Not declared	In this paper authors, examine the origins of social determinants of health & structural violence "tracing their 'prehistory' and little-recognised intersections, based on searches of both concepts" to "examine their similarities and differences, and their potentialities and limitations."	People, places	N/A	N/A	X

Year	Author	Authorship	Framework/ document title	Source	Type of document	Funding source	Purpose (overarching aim)	Components	Levers & enablers	Levels	Res. Ag
**2020**	Raphael et al.[22]	HIC	Social Determinants of Health: The Canadian Facts, 2nd Edition	Google scholar	Book	Ontario Tech University Faculty of Health Sciences	Introduction to the key social determinants of health and contributed significantly to shifting our thinking about what contributes to health and health inequities and what we can do to promote health and reduce these health equities. How living conditions "get under the skin" to either promote health or cause disease.	People, places, planet, products	RFM IA RS PM GK	Country, global	✓
**2020**	United Nations [40]	INT	UN Research Roadmap for the COVID-19 Recovery: Leveraging the Power of Science for a More Equitable, Resilient and Sustainable Future	Added by authors	Research roadmap	UN	This document aims to inform the "recovery efforts" to address challenges from COVID-19, and "sets out the framework for the UN family's urgent socio-economic support to countries and societies across the globe." This document is "a tool that can be used by researchers, research funding agencies, civil society organizations, governments and international institutions to build partnerships, align research response efforts, and demonstrate the power of global science."	People	RFM IA PWA PM GK	Country	✓
**2020**	United Nations Development Programme[19]	INT	Human Development Report 2020: The next frontier Human development and the Anthropocene	Added by authors	Report	UNDP	This document states it provides a "thought-provoking, necessary alternative to paralysis in the face of rising poverty and inequalities alongside alarming planetary change". It presents the new, experimental Planetary pressures–adjusted Human Development Index.	People, planet, places, products	RFM IA RS PWA PM GK	Global, region, country, community	✓
**2021**	Lacy-Nichols & Marten[91]	HIC	Power and the commercial determinants of health: ideas for a research agenda	Added by authors	Journal article	No funding	This commentary paper argues for a "power lens" to better examine "the sources and consequences of corporate actors' market and political influence, [and] opportunities to challenge or diminish this power."	Products	RFM	N/A	✓
**2021**	US House of Representatives [41]	HIC	A Bold Vision for a Legislative Path Toward Health and Economic Equity	Added by authors	Committee Report	House Committee on Ways and Means	The Foreword of this report it states "the American Dream begins and ends with economic opportunity, but that opportunity is not distributed evenly across the nation. For many people of color, good jobs with good pay are disproportionately out of reach. The framework we present here is Ways and Means Committee Democrats' plan to make our nation a more just and equitable place."	People, places	RFM IA RS PM	Country, community	X

Note: For 'Components' and 'Levels', items are listed in order of prominence in the document.

Key

Authorship: affiliations for authors of each publication were reviewed and categorised as high income country (HIC), low and middle income country (LMIC), international or regional entity (INT) or not specified.

Levers and enablers RFM-Regulatory and fiscal measures; IA- Intersectoral action, RS- Redefining "success", PWA- Political will and accountability, PM- Popular mobilisation; GK- Generation and use of knowledge

Res Ag: whether the document had a research agenda

Excluded papers

Source	Documents excluded
Maani et al. - Total references in original paper (n=34)<br>• Total extracted based on title and abstract (n = 12)<br>o Total retained in sample (n = 8)<br>o Total excluded (n = 4)	1. Bircher J, Hahn EG. Will the Meikirch model, a new framework for health, induce a paradigm shift in healthcare? Cureus 2017;9:e1081<br>2. Fisher-Owens SA, Gansky SA, Platt LJ, et al. Influences on children's oral health: a conceptual model. Pediatrics 2007;120:e510–20.<br>3. Huynen MM, Martens P, Hilderink HB. The health impacts of globalization: a conceptual framework. Global Health 2005;1:14.<br>4. Moss NE. Gender equity and socioeconomic inequality: a framework for the patterning of women's health. Soc Sci Med 2002;54:649–61.
Van Olmen et al. - Total references in original paper (n=88)<br>• Total extracted based on title and abstract (n = 5)<br>o Total retained in sample (n = 4)<br>o Total excluded (n=1)	5. Halstead S, Walsh J, Warren K, 1985. Good health at low cost. Rockefeller Foundation, Bellagio.
Google Scholar search<br>• Total extracted based on title and abstract (n=35)<br>o Total retained in sample (n =31)<br>o Total Excluded (n=4)	6. Eriksson, M., 2011. Social capital and health–implications for health promotion. Global health action, 4(1), p.5611.<br>7. Ståhl, T., Wismar, M., Ollila, E., Lahtinen, E. and Leppo, K. eds., 2006. Health in all policies: prospects and potentials.<br>8. Starfield, B., 2001. Basic concepts in population health and health care. Journal of Epidemiology & Community Health, 55(7), pp.452-454.<br>9. World Health Organization. Regional Office for the Eastern Mediterranean. (2002) .Health and human security .
Documents identified through snowballing and author suggestion- (n=45)<br>• Total extracted from (n = 45)<br>o Total retained in sample (n =25)<br>o Total Excluded (n=20)	10. Angkurawaranon, C., Jiraporncharoen, W., Chenthanakij, B., Doyle, P. and Nitsch, D., 2014. Urbanization and non-communicable disease in Southeast Asia: a review of current evidence. Public health, 128(10), pp.886-895.<br>11. Bogar, S. and K.M. Beyer, Green Space, Violence, and Crime: A Systematic Review. Trauma Violence Abuse, 2016. 17(2): p. 160-171.<br>12. Centers for Disease Control and Prevention, 2002, The Social-Ecological Model: A Framework for Violence Prevention. Health, 1, p.56.<br>13. Clark, D.A., 2005. Sen's capability approach and the many spaces of human well-being. The Journal of Development Studies, 41(8), pp.1339-1368.<br>14. De Leeuw, E., Green, G., Dyakova, M., Spanswick, L. and Palmer, N., 2015. European Healthy Cities evaluation: conceptual framework and methodology. Health promotion international, 30(suppl_1), pp.i8-i17.<br>15. The Harare Commonwealth Declaration, 1991<br>16. Heath, G.W., et al., The Effectiveness of Urban Design and Land Use and Transport Policies and Practices to Increase Physical Activity: A Systematic Review. J Phys Act Health, 2006. 3(s1): p. S55-S76.<br>17. Smith, M., Hosking, J., Woodward, A., Witten, K., MacMillan, A., Field, A., Baas, P. and Mackie, H., 2017. Systematic literature review of built environment effects on physical activity and active transport–an update and new findings on health equity. International journal of behavioral nutrition and physical activity, 14(1), pp.1-27.<br>18. Tobgay, T., Dophu, U., Torres, C.E. and Na-Bangchang, K., 2011. Health and Gross National Happiness: review of current status in Bhutan. Journal of Multidisciplinary Healthcare, pp.293-298.<br>19. World Health Organization & WHO Centre for Health Development (Kobe, Japan() .2010). Urban HEART: Urban Health Equity Assessment and Response Tool. World Health Organization<br>20. World Health Organization. Division of Operational Support in Environmental Health. Urban Environmental Health Unit & WHO Healthy Cities Programme. (1995(.WHO Healthy Cities :a programme framework ,a review of the operation and future development of the WHO Healthy Cities Programme /prepared by the Unit of Urban Environmental Health, Division of Operational Support in Environmental Health. World Health Organization.
