# Supplementary material for: What Are Healthy Societies? A Thematic Analysis of Relevant Conceptual Frameworks
Source: Int J Health Policy Manag. 2023 Nov 7;12:7450. doi: 10.34172/ijhpm.2023.7450 (PMC10699824; doi:10.34172/ijhpm.2023.7450)
Supplement: Supplementary file 2 — Data Extraction Template. [file ijhpm-12-7450-s002.pdf]

Article title: What Are Healthy Societies? A Thematic Analysis of Relevant Conceptual Frameworks

Journal name: International Journal of Health Policy and Management (IJHPM)

Authors' information: Kent Buse[1]*, Amy Bestman[2], Siddharth Srivastava[3], Robert Marten[4], Sonam Yangchen[4], Devaki Nambiar[3,2,5]

[1]The George Institute for Global Health, Imperial College London, London, UK.

[2]Faculty of Medicine, University of New South Wales, Sydney, NSW, Australia.

[3]The George Institute for Global Health, New Delhi, India.

[4]The Alliance for Health Policy and Systems Research, World Health Organization (WHO), Geneva, Switzerland.

[5]Prasanna School of Public Health, Manipal Academy of Higher Education, Manipal, India.

***Correspondence to:** Kent Buse; Email: kentbuse@gmail.com

Citation: Buse K, Bestman A, Srivastava S, Marten R, Yangchen S, Nambiar D. What are healthy societies? A thematic analysis of relevant conceptual frameworks. Int J Health Policy Manag. 2023;12:7450. doi:10.34172/ijhpm.2023.7450

Supplementary file 2. Data Extraction Template

Data field	Definition
Document	Document title
Source	Where the document was source from
Date	Date published
Overarching aim(s)	As framed by authors - what the document/initiative intends to do
Authors	-
Author Affiliations	-
Approach/ Process followed	Short form of what kind of process the document represents, e.g., conference declaration, commission report, research/systematic review
Funded by	Entity or institution that has funded the work/donor
Broad topics/ thematic area(s)	As framed by the authors - what are the main things the document is conveying - 1) where there were section headings these were seen as themes, 2) also keywords (in abstracts); 2) what emerged in reading - 3) from the title, 4) where a framework has been defined/specified
Policy Levers/ Stakeholders implicated	Word search for "policy" or "agenda" or "planning" - refers to what government has used to implement something, includes policy decisions (often upstream, or outside the health sector)
Key Takeaways/ Recommendations	Text under headings like "recommendations or key insights;" also, examples or case studies that offer more lessons
Research agenda advocated	Word search for "research" or "researchers" or "academia" - there usually is a section dedicated to this - defined by actors or by knowledge gaps
Values and principles	Extract data on how the paper thinks/approaches/views an issue/what is important
Power	Word search for "power" or "empowerment", also historical references to stakeholders and processes - Usually towards the end, key stakeholders and agents in the document and relations between them or the agency that they have
Related	Reference to other documents that are critically linked to, or part of the document being reviewed
Comments	Extractor/Researcher reflections based on the document
