# Supplementary material for: What Are Healthy Societies? A Thematic Analysis of Relevant Conceptual Frameworks
Source: Int J Health Policy Manag. 2023 Nov 7;12:7450. doi: 10.34172/ijhpm.2023.7450 (PMC10699824; doi:10.34172/ijhpm.2023.7450)
Supplement: Supplementary file 4 — Values Present in the Sample Documents. [file ijhpm-12-7450-s004.pdf]

Article title: What Are Healthy Societies? A Thematic Analysis of Relevant Conceptual Frameworks

Journal name: International Journal of Health Policy and Management (IJHPM)

Authors' information: Kent Buse[1]*, Amy Bestman[2], Siddharth Srivastava[3], Robert Marten[4], Sonam Yangchen[4], Devaki Nambiar[3,2,5]

[1]The George Institute for Global Health, Imperial College London, London, UK.

[2]Faculty of Medicine, University of New South Wales, Sydney, NSW, Australia.

[3]The George Institute for Global Health, New Delhi, India.

[4]The Alliance for Health Policy and Systems Research, World Health Organization (WHO), Geneva, Switzerland.

[5]Prasanna School of Public Health, Manipal Academy of Higher Education, Manipal, India.

***Correspondence to:** Kent Buse; Email: kentbuse@gmail.com

Citation: Buse K, Bestman A, Srivastava S, Marten R, Yangchen S, Nambiar D. What are healthy societies? A thematic analysis of relevant conceptual frameworks. Int J Health Policy Manag. 2023;12:7450. doi:10.34172/ijhpm.2023.7450

Supplementary file 4. Values Present in the Sample Documents

Date	Author	Equity	Collaboration	Justice	Equality	Access	Empowerment	Human rights	Wellbeing driven	Sustainability	Partnerships	Capacity building	Power	Inclusion	Fairness	Governance	Self-determination	Culture preserving	Conservation	De-colonising	Democracy	Diversity
		**39**	**16**	**14**	**14**	**13**	**13**	**11**	**10**	**9**	**9**	**9**	**8**	**7**	**6**	**6**	**4**	**4**	**3**	**2**	**2**	**1**
1975	Lalonde	✓				✓																
1978	WHO	✓	✓	✓	✓	✓		✓			✓	✓		✓			✓					
1986	WHO Euro	✓	✓	✓	✓		✓			✓	✓							✓	✓			
1993	Sen	✓			✓		✓					✓	✓		✓							
1996	Hamilton & Bhatti	✓		✓																		
2000	Berkman et al.																					
2000	People 's Health Movement	✓	✓				✓	✓														
2001	Participants of the conference on "Health Care for All"	✓	✓			✓						✓			✓							
2003	Chen & Narasimhan		✓		✓			✓														

2003	Ogata & Sen	✓		✓	✓	✓	✓	✓	✓		✓	✓	✓			✓	✓				✓	
2004	Schulz & Northridge				✓																	
2005	Gasper	✓						✓					✓	✓								
2006	Barton & Grant								✓	✓												
2007	Dahlgren& Whitehead	✓	✓	✓		✓		✓							✓							
2006	Etches et al.			✓																		
2006	Ståhl et al.		✓								✓										✓	
2006	Whitehead & Dahlgren	✓		✓		✓	✓	✓					✓		✓							
2007	Siddiq et al.	✓									✓											
2007	Whitehead	✓																				
2008	Commission on Social Determinants of Health	✓	✓	✓		✓									✓							
2008	Dyck								✓								✓	✓		✓		
2008	Hiatt & Breen																					
2008	Kawachi et al.								✓					✓								
2009	Fox & Meier			✓	✓			✓														
2010	Bambra et al.				✓																	
2010	Bozorgmehr												✓									
2010	Solar & Irwin	✓		✓	✓		✓	✓														
2011	Braveman et al.																					
2012	Golden & Earp								✓													
2012	Lorenc et al.					✓			✓					✓								
2013	Board on Population Health Public Health Practice et al.	✓				✓	✓															
2013	WHO Regional Committee for Europe		✓		✓		✓				✓	✓				✓						
2014	Krumeich & Meershoek	✓																				
2015	Welsh et al.	✓							✓													
2014	WHO	✓	✓	✓						✓		✓		✓								
2015	Ball et al.	✓																				
2015	Friel et al.	✓																				
2015	Newman et al.	✓																				
2015	Purcell	✓																				
2015	Roche et al.	✓																				
2015	VicHealth[a]	✓									✓				✓							
2015	VicHealth[b]	✓																				
2015	VicHealth[c]	✓																				
2015	Whitmee et al.	✓		✓						✓						✓						
2016	Attendees of the 9th Global Conference on Health Promotion	✓					✓		✓													
2016	Graham & White																					
2017	Buse et al.																					
2017	Boswell et al.		✓	✓	✓																	

2017	McNamara																					
2018	Koehler		✓													✓						
2018	Kondo									✓		✓										
2018	WHO Independent High-level Commission on NCDs	✓					✓	✓			✓	✓										
2018	Vik & Carlquist								✓													
2019	Cerf	✓				✓																
2019	New Zealand Treasury	✓	✓			✓	✓		✓		✓			✓				✓				
2019	Swinburn et al.																					
2019	Verma	✓								✓			✓			✓		✓	✓			
2019	WHO	✓	✓		✓	✓		✓				✓				✓						
2018	Willett et al.									✓												
2020	Amuasi et al.		✓							✓									✓			
2020	Hawkes & Buse	✓			✓		✓															
2020	Herrick and Bell				✓																	
2020	Raphael et al.	✓		✓																		
2020	United Nations	✓								✓												
2020	United Nations Development Programme												✓									
2021	Lacy-Nichols & Marten												✓									
2021	US House of Representatives	✓	✓			✓	✓							✓			✓			✓		✓
